# Left-Sided Amyand’s Hernia: A Rare Variant of Inguinal Hernia

**DOI:** 10.7759/cureus.45113

**Published:** 2023-09-12

**Authors:** Khalid Alyahyawi

**Affiliations:** 1 Department of Surgery, Faculty of Medicine, Jazan University, Jazan, SAU

**Keywords:** inguinoscrotal hernia, inguinal hernia, case report, rare form inguinal hernia, amyand’s hernia

## Abstract

Inguinal hernia is the most commonly diagnosed hernia, with approximately one out of every three males eventually being diagnosed with an inguinal hernia. Amyand's hernia is a subtype of an inguinal hernia that occurs when the appendix is located within the hernia sac. It is an uncommon condition that is usually discovered as an incidental finding in less than 1% of all patients with inguinal hernia. The management options for this condition will vary depending on the presence or absence of appendicitis. This case report highlights a rare occurrence where a patient with a left-sided scrotal swelling was found to have a left-sided Amyand's hernia that was eventually managed without complications.

## Introduction

Amyand's hernia is an uncommon variant of inguinal hernia where the appendix resides within the hernia sac. It is named after Claudius Amyand, the surgeon who first identified this condition [1]. The incidence of Amyand's hernia is estimated to be 1% of all inguinal hernias. Usually, it is discovered as an incidental finding intraoperatively [2]. However, its presentation and management options may vary according to the presence or absence of inflammation, i.e., uncomplicated appendicitis or other complications like perforation or an abscess. The presence of a left-sided Amyand's hernia is even more rare, accounting for 9.5% of patients with Amyand's hernias [3] mostly due to the anatomical location of the appendix. In this article, we report a case of an 81-year-old male who presented with a large left-sided inguinal hernia, which was identified intraoperatively as an Amyand's hernia. A brief review of the clinical presentation, diagnosis, and management of this case of Amyand's hernia is discussed.

## Case presentation

**Figure 1:**
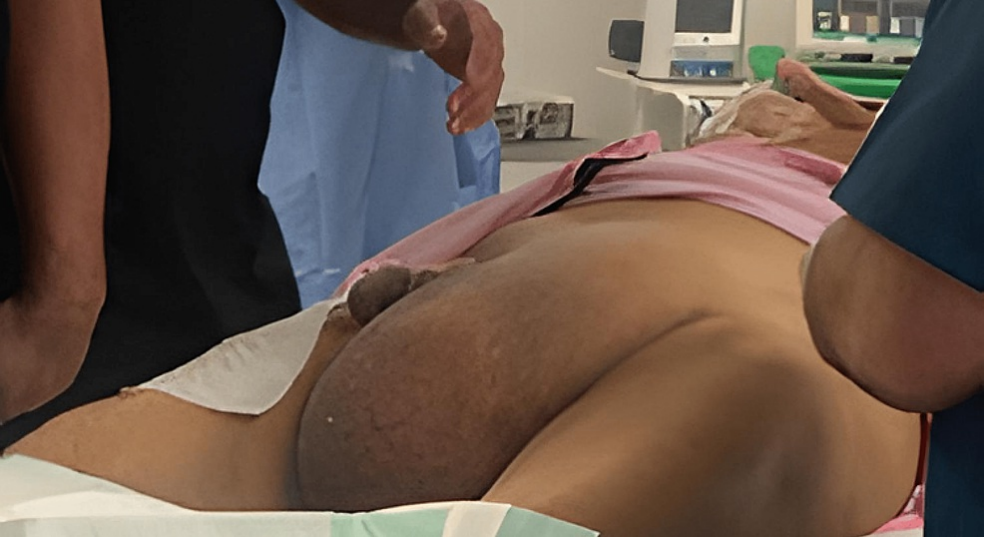
Left-sided inguinoscrotal swelling of the patient

An 81-year-old male patient known to have diabetes mellitus type 1 on insulin therapy and arterial hypertension with no history of prior abdominal surgeries presented with a scheduled appointment to the surgical outpatient clinic with a longstanding history of left-sided inguinal swelling and a history of pain that was progressively worsening in the last four days before presentation (Figure 1). The patient did not complain of nausea or vomiting, nor did he complain of any urinary or other gastrointestinal symptoms. A physical examination revealed a left inguinoscrotal hernia, which was not reducible. The overlying skin appeared inflamed but intact. Laboratory investigations did not reveal any abnormalities. Ultrasound examination revealed a left inguinal hernia with a thickened hernia sac with intestinal content.

**Figure 2:**
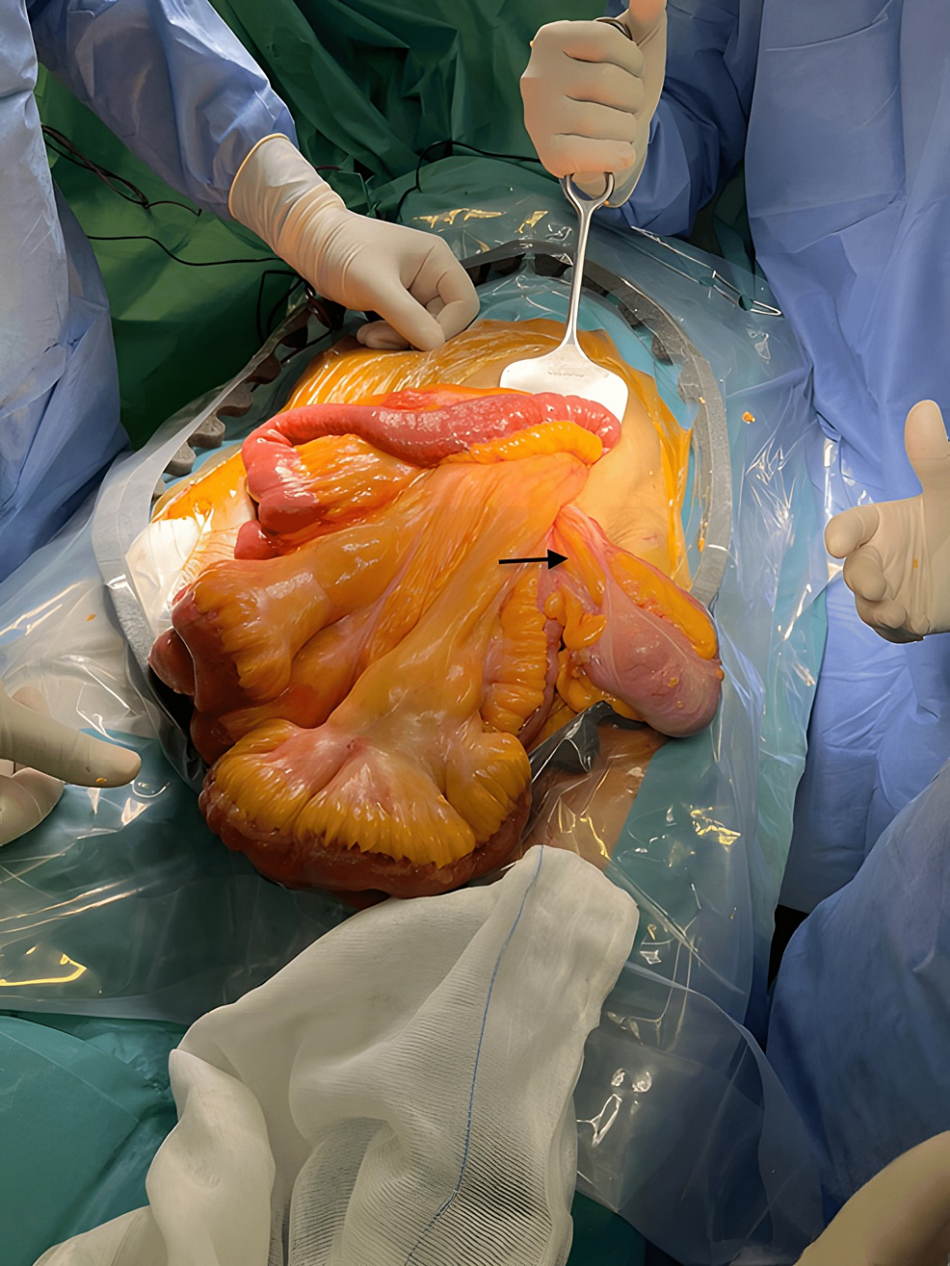
Intraoperative photograph showing a loop of the terminal ileum, cecum, and appendix inside the hernia

The patient underwent a Lichtenstein tension-free mesh hernia repair, during which a suction drain was placed and spinal anesthesia was administered. The intraoperative examination revealed a large, approximately 30 cm by 15 cm left-sided hernia containing a loop of the terminal ileum, cecum, and appendix (Figure 2).

The postoperative course was uneventful. The drain was removed on the second postoperative day and the patient was discharged without complications. The patient was followed up in the surgical clinic and remained asymptomatic six months postoperatively.

## Discussion

Amyand's hernia is a rare form of inguinal hernia that was first described by Claudius Amyand in 1735. The appendix, which is normally located in the right lower quadrant of the abdomen, can herniate through the inguinal canal and be incarcerated within the hernia sac. The incidence of Amyand's hernia is estimated to be 1% of all inguinal hernias [2] though only 0.1% of cases complicate into acute appendicitis due to late presentation and missed diagnosis [4].

The clinical presentation of Amyand's hernia is highly variable and depends on whether the appendix is inflamed or not. An inflamed appendix within the hernia sac can cause acute appendicitis, which presents as acute abdominal pain, fever, nausea, vomiting, and leukocytosis; in some cases, it may lead to incarceration, strangulation, necrosis, perforation, or rupture. Early symptoms include tenderness and inguinal swelling, which may be misdiagnosed as a strangulated hernia. This condition can be difficult to diagnose clinically [1,5].

Imaging studies, such as ultrasound and CT scans, can aid in the diagnosis of Amyand's hernia. Ultrasound can detect the presence of an inguinal hernia and identify the appendix within the hernia sac. A CT scan can provide high-resolution images of the hernia sac and the appendix and can differentiate between an inflamed and non-inflamed appendix [2].

The management of Amyand's hernia depends on the clinical presentation and whether the appendix is inflamed or not. In case of an inflamed appendix, early surgical intervention is recommended to prevent complications such as perforation, abscess, and sepsis. An appendectomy can be performed either through an open or laparoscopic approach, depending on the surgeon's preference and the patient's condition. The hernia can be repaired simultaneously with appendectomy using either a mesh or non-mesh technique, depending on the surgeon's preference and the patient's risk factors [1]. The use of the Losanoff and Basson classification system to identify and manage Amyand's hernias will allow surgeons to make informed decisions regarding the treatment of these conditions. The choice of whether to remove or keep a non-inflamed appendix depends on the patient's age, general health conditions, and tolerance. The classification is described in Table 1 [5-8].

**Table 1:** Losanoff and Basson's classification Adapted from [1].

Classification	Description	Surgical management
Type 1	Normal appendix within an inguinal hernia	Hernia reduction, mesh repairs, appendectomy in young patients
Type 2	Acute appendicitis within hernia, no abdominal sepsis	Appendectomy through hernia primary repair of hernia, no mesh
Type 3	Acute appendicitis within an inguinal hernia, abdominal wall, or peritoneal sepsis	Laparotomy, appendectomy, primary repair of hernia, no mesh
Type 4	Acute appendicitis within an inguinal hernia, related or unrelated abdominal pathology	Manage as a type 1-3 hernia and investigate or treat the second condition as appropriate

## Conclusions

Amyand's hernia is a rare form of inguinal hernia that can pose a diagnostic and therapeutic challenge. It usually occurs in the right inguinal region. A high degree of clinical suspicion and prompt radiological investigation can aid in the diagnosis of Amyand's hernia. However, as shown above, it may present on the left inguinal region in patients presenting with large inguinoscrotal hernias. Early surgical intervention is recommended, especially in cases of an inflamed appendix, to prevent complications and ensure a good outcome.

## References

[1] Losanoff JE, Basson MD (2008). Amyand hernia: a classification to improve management. Hernia.

[2] Green J, Gutwein LG (2013). Amyand's hernia: a rare inguinal hernia. J Surg Case Rep.

[3] Manatakis DK, Tasis N, Antonopoulou MI (2021). Revisiting Amyand's hernia: a 20-year systematic review. World J Surg.

[4] Faiz N, Ahmad N, Singh R (2016). Case series on different presentations of Amyand’s hernia. Arch Int Surg.

[5] Michalinos A, Moris D, Vernadakis S (2014). Amyand's hernia: a review. Am J Surg.

[6] Michalinos A, Moris D, Vernadakis S (2015). Amyand's hernia: a case series with critics of role of appendectomy. Hernia.

[7] Morales-Cárdenas A, Ploneda-Valencia CF, Sainz-Escárrega VH, Hernández-Campos AC, Navarro-Muñiz E, López-Lizarraga CR, Bautista-López CA (2015). Amyand hernia: case report and review of the literature. Ann Med Surg (Lond).

[8] Yagnik VD (2011). Amyand hernia with appendicitis. Clin Pract.

